# From Birth to Unintended Pregnancy: Postnatal Contraception Failures in the First Postpartum Year

**DOI:** 10.7759/cureus.105523

**Published:** 2026-03-19

**Authors:** Maryam Javed, Falak Naz Baloch, Vandna Verma

**Affiliations:** 1 Department of Obstetrics and Gynaecology, Bedfordshire Hospitals NHS Foundation Trust, Bedford, GBR; 2 Department of Obstetrics and Gynaecology, Milton Keynes University Hospital NHS Foundation Trust, Milton Keynes, GBR

**Keywords:** healthcare proffesionals, patient education, postnatal contraception, postpartum family planning, termination of pregnancy, unintended pregnancy

## Abstract

Background: In the postpartum period, fertility and sexual activity can return rapidly, placing women at risk of unintended pregnancy within the first 12 months following delivery. Unintended pregnancies during this period, particularly with short interpregnancy intervals, are associated with adverse maternal and neonatal outcomes. Postnatal contraception (PNC) counselling is often inconsistent, and gaps exist in both patient and provider knowledge and practices.

Objective: The objective of this study is to assess knowledge, attitudes, and practices of healthcare professionals and postnatal women regarding PNC and to evaluate the termination of pregnancy (TOP) clinic to review women seeking termination within 12 months of delivery in a UK district general hospital.

Methods: A cross-sectional, questionnaire-based survey of healthcare professionals and postnatal women was conducted between September 2024 and December 2024. In parallel, a retrospective study of women undergoing TOP between December 2023 and November 2024 was performed using electronic medical records. Quantitative survey data were analysed descriptively, and free-text responses were thematically analysed.

Results: Fifty-seven healthcare professionals (73.7% midwives (n= 42/57), 26.3% (n= 15/57) doctors) and 60 postnatal women participated. All staff acknowledged the importance of PNC counselling, but midwives were more likely than doctors to view it as part of their role (83.3% (n= 35/42) vs 40% (n= 6/15)). Knowledge of early return of fertility was limited (26.6% (n= 4/15) of doctors; 7.1% (n= 3/42) of midwives). Common barriers included time constraints and workload, and most staff expressed interest in further training (92.8% (n= 39/42) of midwives; 80% (n= 12/15) of doctors), preferring online modules 58% (n= 33/57) or face-to-face sessions 28% (n= 16/57).

Among women, 53.4% (n= 32/60) were multiparous, 43.3% (n= 26/60) reported the recent pregnancy as unplanned, only 26.6% discussed PNC postnatally, mostly verbally, and awareness of fertility returning as early as three weeks postpartum was low (13.3%; n= 8/60). The audit showed that 15.4% (n= 29 of 188) women underwent TOP within 12 months of delivery, indicating that approximately one in five women experienced unintended pregnancies shortly after childbirth.

Conclusion: Significant gaps exist in knowledge and counselling practices regarding PNC among both healthcare professionals and women. The findings underscore the need for systematic staff training and structured counselling pathways to improve timely uptake of contraception, reduce short interpregnancy intervals, and prevent unintended postpartum pregnancies.

## Introduction

In the postpartum period, fertility and sexual activity can return rapidly, placing women at risk of unintended pregnancies in the 12 months following a delivery [1]. As recommended by the World Health Organization (WHO), there should be a minimum interval of 24 months from delivery to the conception of the next pregnancy to minimise the risk of negative outcomes for mothers, newborns and infants [2]. Effective postpartum contraception is essential for preventing unintended pregnancies and their associated consequences [3]. Unintended pregnancy during the postpartum period, particularly with interpregnancy intervals of less than 12 months, is associated with increased risks of adverse maternal and neonatal outcomes, including maternal anemia, hypertensive disorders, and postpartum complications as well as neonatal complications including preterm birth, fetal growth restriction, and stillbirth [3].

The Faculty of Sexual and Reproductive Healthcare (FSRH) recommends discussing contraception during pregnancy to ensure that the chosen method can be provided immediately postpartum, before hospital discharge [4,5]. The postpartum period represents a critical window for contraceptive counselling, as fertility may return as early as three weeks following delivery, even in women who are breastfeeding [5]. Failure to timely provide postnatal contraception (PNC) counselling results in missed opportunities for pregnancy prevention, with nearly one in 13 women in our population conceiving within one year of delivery [6].

A study from Tanzania reported that women who received family planning counselling during postnatal care visits were significantly more likely to use contraception than those who did not, highlighting missed opportunities for preventing unintended pregnancies and underscoring the need for better integration of family planning counselling into antenatal and postnatal care services [7].

Despite national and international guidance, PNC counselling practices remain inconsistent, with missed opportunities reported during both antenatal care and postnatal hospital admission [6,7]. Barriers commonly cited include time constraints, competing clinical priorities, and inadequate training among healthcare professionals [8,9].

Unintended pregnancy, defined as a pregnancy that occurs earlier than desired or when no pregnancy is desired, is a significant global public health issue affecting sexually active women [10]. In settings with legal or cultural restrictions on termination of pregnancy (TOP), unintended postpartum pregnancies may have particularly significant consequences, as women may be compelled to continue unplanned pregnancies, increasing the risk of adverse maternal, psychological and social outcomes [11]. Effective postpartum contraception is essential for preventing unintended pregnancies and their associated consequences [12].

This study aimed to assess the knowledge, attitudes, and practices of healthcare professionals and postnatal women regarding PNC in a UK district general hospital, alongside an evaluation of women seeking TOP within 12 months of delivery, to identify barriers to effective counselling and opportunities for service improvement.

## Materials and methods

This study employed a mixed-methods design, comprising a cross-sectional, questionnaire-based survey and service evaluation project, conducted at a United Kingdom (UK) district general hospital from September 2024 to December 2024. Healthcare professionals, including doctors of all grades and midwives involved in maternity care, and postnatal women admitted following delivery were invited to participate in the survey prior to hospital discharge. Women unable to provide informed consent or with significant language barriers without interpreter support were excluded. Participation was voluntary and anonymous, and completion of the questionnaire implied consent.

Two anonymous, self-administered questionnaires were used. The staff questionnaire assessed knowledge regarding return of fertility, attitudes toward PNC, perceived professional responsibilities, barriers to counselling, and training needs. The patient questionnaire collected demographic data, pregnancy planning status, awareness of fertility return, experiences of PNC counselling, and sources of information received. The full questionnaires are provided in Appendix A (staff questionnaire) and Appendix B (patient questionnaire). Quantitative survey data were summarised using descriptive statistics (frequencies and percentages), while free-text responses were analysed thematically.

In parallel, a service evaluation of the TOP clinic was conducted to assess the proportion of women seeking termination within 12 months of delivery, covering a period of one year between December 2023 and November 2024. Data was collected retrospectively from electronic medical records in accordance with institutional policies on confidentiality and data protection. Women were excluded if data were incomplete, if conception occurred while using contraception (except barrier methods), or if the woman was undecided regarding TOP. Data was analysed descriptively using frequencies and percentages to determine patterns relevant to postnatal contraceptive provision.

This study was conducted as a service evaluation, performed with permission and approval of the clinical audit department of the hospital. Participation in the survey was voluntary and anonymous, and informed consent (verbal) was taken prior to completion of the questionnaire as part of the survey. The data collected retrospectively from the TOP clinic did not require patient consent as it was anonymised; hence, no ethical approval was needed except formal registration with the audit department.

## Results

A total of 57 (N=57) healthcare professionals completed the survey. Most respondents were midwives (73.7%; n= 42/57) with doctors comprising 26.3% (n= 15/57) (Table 1). All participants acknowledged the importance of PNC, although only 26.6% (n= 15/57) reported discussing it routinely. Knowledge regarding early return of fertility was variable, with a minority recognizing that fertility may return within three weeks postpartum. Common barriers to counselling included time constraints and lack of training, and the majority of respondents expressed interest in additional education (91.2%; n= 52/57), preferring online modules (56.1%; n= 32/57) or face-to-face sessions (28%; n=16/57). Awareness and experience of postpartum intrauterine contraception (PPIUC) were limited, with only 42.1% (n= 24/57) aware of PPIUC and 24.6% (n= 14/57) having prior experience. Full staff responses are presented in Table 1. Most respondents expressed interest in further education, with 92.8% (n= 39/42) of midwives and 80% (n=12/15) of doctors indicating a preference for additional training. Preferred modalities included online modules (58%; n= 33/57) and face-to-face sessions (28%; n= 16/57). 

**Table 1:** Healthcare professional’s questionnaire responses (n = 57) Responses of healthcare professionals regarding postnatal contraception counselling, training, perceived barriers, and awareness and experience of immediate postpartum intrauterine contraception (PPIUC). Data are presented as number (n) and percentage (%). *Multiple responses permitted. †Percentages calculated among respondents with prior experience of PPIUC.

Variable	Response	n	%
Professional role	Midwives	42	74
	Doctors	15	26
Giving postnatal contraception advice is part of the role	Yes	52	91
	No/Unsure	5	8.8
Postnatal contraception discussed routinely	Yes	35	61
	No/Unsure	22	39
Importance of discussing postnatal contraception	Yes	57	100
Knowledge of return of fertility after delivery	≤6 weeks	23	40
	>6 weeks	22	39
	Do not know	12	21
Timing of postnatal contraception advice	Antenatal	3	5.3
	Postnatal	32	56
	Both	22	39
Received training in postnatal contraception	Yes	24	42
	No	33	58
Barriers to providing contraception advice*	Time constraints	18	32
	Lack of training	15	26
	Not usual practice	6	11
	Not aware of guidance	3	5.3
	No barriers	15	26
Would like more training	Yes	48	84
	No/Unsure	9	16
Preferred training format	Online	32	56
	Face-to-face	16	28
	Email/written	6	11
	Other	3	5.3
Awareness of PPIUC	Yes	24	42
	No	33	58
Previous experience of PPIUC	Yes	14	25
	No	43	75
Received training in PPIUC†	Yes	9	64
	No	5	36

Of 75 postnatal women approached, 60 completed the questionnaire (n = 57 after excluding incomplete responses). The mean age was 32 years, and 61.4% (n=35/57) were primiparous. Less than one-third of women had the opportunity to discuss postnatal contraception prior to delivery, and only 31.6% (n=18/57) received information during pregnancy. Among those who received information, verbal communication was the most common format (75%; n= 43/57) and 12.5% (n= 7/57) received written information. Only 29.8% (n= 17/57) of women had decided on a method of contraception, and the hospital was able to provide the chosen method in 35.3% (n= 20/57) of cases. Knowledge regarding the early return of fertility was low, with 13.3% (n= 8/57) correctly identifying the earliest possible return of fertility. Full patient responses are summarised in Table 2.

**Table 2:** Patient questionnaire responses (n = 57) Demographic characteristics, pregnancy-related factors, and experiences of counselling and decision-making regarding postnatal contraception among survey respondents. Data are presented as number (n) and percentage (%). *Multiple responses permitted. †Percentages calculated among respondents who received information on postnatal contraception during pregnancy. ‡Percentages calculated among respondents who had decided on a method of contraception.

Variable	Response	n	%
Age (years)	≤25	6	11
	26–30	14	25
	31–35	17	30
	36–40	14	25
	>40	6	11
Number of children	0–1	35	61
	≥2	22	39
Planned pregnancy	Yes	26	46
	No	31	54
Opportunity to discuss contraception before delivery	Yes	16	28
	No	41	72
Received information on postnatal contraception during pregnancy	Yes	18	32
	No/Unsure	39	68
Format of information received†	Verbal	13	72
	Written/leaflet	3	17
	Online/website	2	11
Source of information†	Hospital antenatal clinic	6	33
	Community antenatal clinic	5	28
	GP	5	28
	Other (media/home)	2	11
Decided on a method of contraception	Yes	17	30
	No/Undecided	40	70
Hospital able to provide the chosen method‡	Yes	6	35
	No	11	65
Reason for not choosing contraception*	Wanted more time/discuss later	20	50
	Did not want contraception	14	35
	Partner vasectomy	3	7.5
	Not discussed/other	3	7.5
Knowledge of return of fertility after delivery	≤2 weeks	16	28
	3–4 weeks	18	32
	6 weeks	12	21
	≥6 months	3	5.3
	Do not know	8	14

The retrospective service evaluation of the TOP clinic included 188 (n=188) women who underwent TOP during the study period. Of these, 15.4% women (n= 29/188) underwent TOP within 12 months of their last delivery (Table 3).

**Table 3:** Characteristics and outcomes of women undergoing termination of pregnancy (TOP) within 12 months of last delivery Characteristics of women undergoing termination of pregnancy (TOP) within 12 months of a previous delivery, including timing, ethnicity, delivery location, and postnatal contraceptive use. Data are presented as number (n) and percentage (%). Percentages for subcategories are calculated among women who underwent TOP within 12 months postpartum.

Parameter	Number (n)	Percentage (%)
Total number of women who underwent TOP	188	100%
Women with TOP within 12 months postpartum	29	15.40%
Within six months	12	41.40%
Between 6 and 12 months	17	58.60%
Ethnicity of women with TOP <12 months		
White British	19	65.50%
Black African	6	20.70%
Asian	4	13.80%
Delivery location		
Bedford Hospital	18	62.10%
Other facilities	11	37.90%
Contraceptive use since last delivery		
None	25	86.20%
Barrier methods	4	13.80%

**Figure 1:**
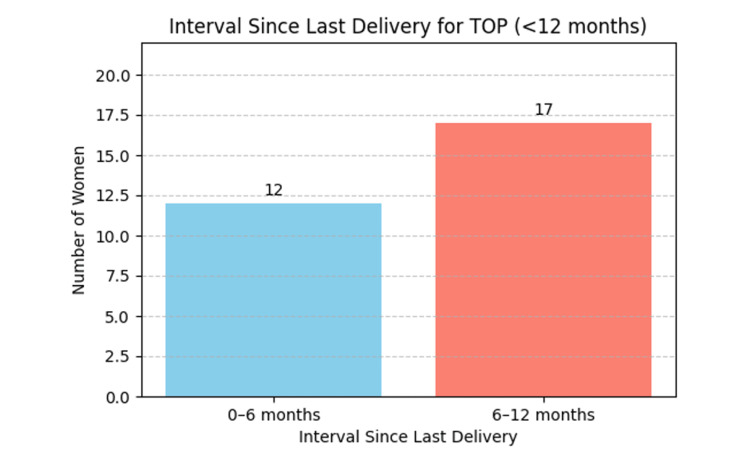
Number of women undergoing termination of pregnancy (TOP) according to the interval since last delivery (within 12 months postpartum)

Among them, 41.4% women (n= 12/29) underwent TOP within six months, and 58.6% women (n= 17/29) between six and 12 months postpartum. The majority were White British (65.5%; n= 19/29), followed by Black African (20.7%; n=6/29) and Asian (13.8%; n= 4/29). Most women delivered their previous baby at Bedford Hospital (62.1%; n= 18/29), while the remainder delivered at other facilities 37.9% (n= 11/29). Regarding contraceptive use since the last delivery, 86.2% (n= 25/29) reported not using any contraception, and 13.8% (n= 4/29) used barrier methods. The distribution of the interval since last delivery is illustrated in Figure 1. These findings highlight the potential consequences of unmet postnatal contraceptive needs and underscore the importance of timely PNC counselling and service provision.

## Discussion

This study demonstrates significant gaps in knowledge, counselling practices, and training related to PNC among both healthcare professionals and postnatal women. Although all healthcare professionals acknowledged the importance of PNC, counselling was inconsistently delivered, and substantial training deficiencies were identified, particularly among midwives, of whom 92.8% (n= 39/42) reported inadequate training. These findings are consistent with existing UK and international literature, which identifies limited training opportunities, time constraints, and competing clinical priorities as key barriers to effective PNC provision [13,14].

Knowledge of early return of fertility postpartum was poor among both healthcare professionals and postnatal women, which is concerning, as inaccurate understanding undermines effective counselling and may contribute to unintended pregnancies. Similar findings have been reported in international studies demonstrating low awareness of lactational amenorrhea as a contraceptive method, emphasizing the need for improved education and strengthened counselling during antenatal and postnatal care. Inadequate provider training and lack of institutional support have consistently been identified as key barriers to effective postpartum contraception provision. Addressing these gaps through structured training, guideline reinforcement, and integration of contraception counselling into routine postnatal pathways may significantly improve outcomes [15].

Only one quarter of women in this study reported receiving PNC counselling, predominantly in verbal form, highlighting missed opportunities to support informed decision-making. Women expressed a desire for clear, early, and consistent advice, preferably delivered during pregnancy or early in the postnatal admission. These findings mirror reports from other maternity settings, where inconsistent counselling and lack of written information contribute to suboptimal contraception uptake [16]. The strong support among healthcare professionals for further education particularly flexible online training suggests that targeted educational interventions may be both feasible and acceptable.

A key finding of this study is the substantial lack of awareness regarding the early return of fertility in the postpartum period among both postnatal women and healthcare professionals. Despite evidence that ovulation can occur as early as three weeks after delivery, particularly in non-breastfeeding women, only a minority of respondents correctly identified this timeframe. This finding is consistent with previous studies demonstrating widespread misconceptions about postpartum fertility and an overestimation of the protective effect of breastfeeding against pregnancy [5,6,15]. The belief that fertility resumes only after the return of menstruation or cessation of breastfeeding may result in delayed uptake of effective contraception and increased vulnerability to unintended pregnancy.

Poor awareness of lactational amenorrhea as a time-limited and criteria-dependent method of contraception has been reported in both low- and high-income settings, highlighting a persistent global knowledge gap [15]. Inadequate understanding among healthcare professionals is particularly concerning, as inaccurate or incomplete counselling may inadvertently reinforce false reassurance among women. Given that many women resume sexual activity before the routine six-week postnatal review, failure to clearly communicate the risk of early fertility return represents a critical missed opportunity for pregnancy prevention and may contribute to the high proportion of unintended pregnancies observed within the first postpartum year [6,14].

Inadequate training was identified as a major barrier to effective PNC counselling in this study, with more than half of healthcare professionals reporting no formal education in this area. Although most respondents acknowledged the importance of PNC, limited knowledge of available methods and lack of confidence, particularly regarding immediate postpartum intrauterine contraception (insertion of devices), appeared to restrict routine counselling. Similar gaps in training and confidence have been reported in studies examining midwifery and medical practice in postpartum family planning, where lack of education and limited institutional support were key barriers to implementation [8,14].

Midwives reported particularly high unmet training needs, which is notable given their central role in providing antenatal and postnatal care. Previous research has shown that midwives who receive targeted training in contraception are significantly more likely to initiate counselling and facilitate uptake of effective methods [8,16]. The strong interest in further education expressed by participants in this study suggests that these barriers are potentially modifiable. Evidence from systematic reviews indicates that structured provider training, combined with clear clinical pathways and guideline reinforcement, can significantly improve postpartum contraceptive counselling and uptake.

Addressing knowledge gaps related to early return of fertility and strengthening healthcare professional training are essential to improving PNC provision. Education on postpartum fertility should be explicitly incorporated into both antenatal and early postnatal counselling, rather than deferred to the six-week review, as recommended by national and international guidance [4,5]. Concurrently, investment in standardized, competency-based training for healthcare professionals-delivered through flexible online modules and supported by face-to-face teaching-may enhance confidence, normalize counselling as routine practice, and reduce missed opportunities for timely contraception provision [9,13,14].

Importantly, evidence indicates that integrating family planning counselling into routine maternal healthcare significantly increases postpartum contraceptive use and reduces unintended and short interpregnancy intervals, thereby improving maternal and child health outcomes. These findings support the need for system-level interventions, including standardized counselling protocols and prioritization of healthcare provider training, to ensure consistent and effective PNC provision [9,13,14].

Strengths of this study include the inclusion of both healthcare professional and patient perspectives, the mixed-methods approach, and the use of real-world data from a busy UK district general hospital, providing insight into routine clinical practice. However, there are some limitations. This was a single-centre study, which may limit generalizability. The reliance on self-reported data introduces the potential for recall or social desirability bias, and the relatively small number of doctors surveyed may not fully represent all staff perspectives. In addition, actual postpartum contraceptive uptake was not assessed, so reported counselling does not necessarily reflect behaviour change. Despite these limitations, the findings are consistent with international literature and reinforce the importance of addressing knowledge and training gaps.

From a practical standpoint, these results emphasise the need for system-level interventions to improve PNC provision. Structured counselling pathways, reinforcement of national guidelines, and standardized protocols can help ensure consistent delivery of information to women. Staff training, including flexible online modules and face-to-face sessions, can enhance confidence and competence in counselling. Providing both written and verbal information and discussing options early in the antenatal and postnatal periods may help reduce missed opportunities, improve timely uptake of contraception, and prevent short interpregnancy intervals. 

## Conclusions

This study highlights significant gaps in PNC knowledge, counselling practices, and staff training among healthcare professionals and postnatal women. The survey revealed low awareness of early return to fertility, inconsistent counselling, and substantial training deficits, particularly among midwives. The audit findings demonstrated that 15.4% (n=29/188) of women underwent TOP within 12 months of delivery, reflecting the potential consequences of unmet postnatal contraceptive needs.

Together, these findings underscore the importance of integrating structured contraception counselling into routine antenatal and postnatal care, strengthening staff training, and providing both written and verbal information to women. Implementing standardised counselling pathways and system-level interventions can improve timely uptake of postpartum contraception, reduce short interpregnancy intervals, prevent unintended pregnancies and ultimately enhance maternal and child health outcomes.

## Appendices

Appendix A: postnatal contraception - staff survey

1.     State your role

·       Midwife (hospital)

·       Community midwife

·       Trainee/registrar/senior clinical fellow

·       Consultant

2.     Do you feel that giving advice on postnatal contraception is part of your clinical role? (Tick)

3.     Is discussing postnatal contraception part of your routine practice?

·       Yes

·       No

·       If no, please mention the reasons -

4.     Do you think that discussing postnatal contraception is important? (tick)

5.     From your knowledge, how quickly can fertility return after delivery?

·       After one week

·       After two weeks

·       After three weeks

·       After four weeks

·       After six weeks

·       After six months

·       After one year

·       Do not know

6.     When do you give postnatal advice to women?

·       Antenatally

·       Postnatally

·       Both

·       Neither

7.     Which methods of contraception do you think are available at Bedford for postnatal women? (tick all available)

·       Progesterone only pill (Minipill)

·       Combined pill

·       Depot injection

·       Nexplanon (implant)

·       Copper coil at caesarean section

·       Mirena coil at caesarean section

·       Coil after vaginal delivery

·       Not sure

8.     Have you received any training or education in postnatal contraception?

·       Yes

·       No

·       If yes, please state all training (courses/e-learning, self-directed learning) -

9.     Is there anything preventing you from giving postnatal contraception advice to women in your care?

·       No

·       Yes

·       If yes, please give the reasons (Tick all applicable)

o   Not the usual practice

o   Not enough time in clinic to discuss PNC (time-constraint)

o   Felt did not have the necessary training

o   Other reasons (please specify)

10.  Would you like more training on postnatal contraception?

·       Yes

·       No

·       Unsure

11.  What format would you like the training in?

·       Online

·       F2F

·       An email signposting me to useful resources and guides

·       Others - please specify

·       I do not want any training (please give a reason) -

12.  Have you heard about PPIUC (postpartum intrauterine contraception)?

13.  Do you have any previous experience in PPIUC?

14.  If previous experience, did you receive any training in PPIUC?

·       Yes

·       No

·       If yes, please mention where you received the training and how?

Thank you for completing this survey!

Appendix B: patient questionnaire

Postnatal ward questionnaire for women - Contraception choices after giving birth

1.     Please state your age:                years

2.     Please state how many children you have:

3.     Please state the gap between your children.                                                               

·       2nd

·       3rd

·       4th

·       5th

4.     Was this a planned pregnancy?

·       Yes

·       No

·       If yes, what contraception were you using prior to this pregnancy?

5.     Did you have an opportunity to discuss contraception with a doctor or your midwife before your baby was born?

·       Yes

·       No

·       Unsure

6.     During pregnancy, did you receive any information on different contraceptive choices you have after delivery?

·       Yes

·       No

·       Unsure

7.     If you did receive this information, in which form was this information given to you?

·       Verbal

·       Information leaflet

·       Directed to a website

·       Combined (state which) -                                                                                        .

8.     If you did receive information on contraceptive choices in pregnancy, where did you receive this advice?

·       Antenatal clinic - hospital

·       Antenatal clinic - community

·       Antenatal class

·       Other (please state) -                                                                                                            .

9.     Did you decide on a method of contraception that you would like to have?

·       Yes - please state what                                                                                                        .

·       No

·       Unsure

10.  Has the hospital been able to provide you with your chosen method of contraception?

·       Yes

·       No - please state why not                                                                                          .

·       I am still undecided

·       I do not want any contraception

·       I would like to get it from my GP/community service

·       Other - please state                                                                                                     .

11.  If you have not yet chosen a method of contraception, please state why (till all applicable)

·       I do not want any contraception

·       I would like to discuss it with my family first

·       I would like to decide at a later date

·       I need time to think

·       Other - please state                                                                                                     .

12.  From your knowledge, when do you think fertility returns after having a baby (the earliest point at which you might be able to get pregnant again after having a baby)?

·       After one week

·       After two weeks

·       After three weeks

·       After four weeks

·       After six weeks

·       After six months

·       After one year

·       Do not know

Thank you so much for completing this survey!

We are very grateful for your participation.

Please return this questionnaire to a member of the staff.
